# The genome sequence of the small skipper, *Thymelicus sylvestris *(Poda, 1761)

**DOI:** 10.12688/wellcomeopenres.17577.1

**Published:** 2022-02-01

**Authors:** Alex Hayward, Ryan Biscocho, Darwin Tree of Life Barcoding collective, Wellcome Sanger Institute Tree of Life programme, Wellcome Sanger Institute Scientific Operations: DNA Pipelines collective, Tree of Life Core Informatics collective, Darwin Tree of Life Consortium

**Affiliations:** 1 Department of Biosciences, University of Exeter, Penryn, TR10 9FE, UK

**Keywords:** Thymelicus sylvestris, small skipper, genome sequence, chromosomal, Lepidoptera

## Abstract

We present a genome assembly from an individual male *Thymelicus sylvestris* (the small skipper; Arthropoda; Insecta; Lepidoptera; Hesperiidae). The genome sequence is 471 megabases in span. The majority of the assembly (99.97%) is scaffolded into 27 chromosomal pseudomolecules, with the Z sex chromosome assembled. The mitochondrial genome was also assembled and is 17.1 kilobases in length.

## Species taxonomy

Eukaryota; Metazoa; Ecdysozoa; Arthropoda; Hexapoda; Insecta; Pterygota; Neoptera; Endopterygota; Lepidoptera; Glossata; Ditrysia; Hesperioidea; Hesperiidae; Hesperiinae; Hesperiini; Thymelicus; *Thymelicus sylvestris* (Poda, 1761) (NCBI:txid272628).

## Background

The small skipper ( *Thymelicus sylvestris*) is a butterfly within the skipper family Hesperiidae. The skippers are named for their characteristic quick, darting flight. The common name of *T. sylvestris* is a clear reference to its small size: the adult wingspan ranges from 27–34 mm ( Tomlinson & Still, 2002). However, it is not the smallest of the skippers, with four other British species being an equivalent size or smaller. Similar to other skippers, *T. sylvestris* has golden-orange wings with clear sex brands on males, but it can be distinguished by a lack of coloured patches on its wings and a dull brown or orange colouration to its antennae ( Tomlinson & Still, 2002).

*Thymelicus sylvestris* is widespread across the European continent with a habitat range paralleling that of other skipper species. This range encompasses the northernmost reaches of Morocco and Algeria all the way to the bordering regions between the Baltic states and Russia ( Tolman & Lewington, 2008). However, it is noticeably absent from northern Scandinavia, Corsica and Sardinia ( Tolman & Lewington, 2008). In the British Isles the small skipper is found across most of Wales and England with recent trends showing a northward expansion in range, beyond the England-Scotland border. Recently, *T. sylvestris* individuals have also been observed in Ireland, where they had not been reported previously ( Harding & Jacob, 2013). *Thymelicus sylvestris* populations appear stable and it is listed as a species of least concern by the IUCN ( van Swaay *et al.*, 2010).

The small skipper is a habitat generalist ( Louy *et al.*, 2007) and can be found in open areas with long grass, such as rough grasslands and roadside verges ( Tomlinson & Still, 2002). It is most associated with Yorkshire fog ( *Holcus lanatus*), its main food plant, on which it often basks and lays eggs from June to July. Females are known to be meticulous with their choice of oviposition sites, spending up to 15 minutes inspecting potential host plants prior to laying eggs ( Tolman & Lewington, 2008). After approximately a month, eggs hatch into caterpillars which develop through 5 instar stages. Come winter, caterpillars spin cocoons within which they undergo diapause. The caterpillars re-emerge in spring, constructing a ‘leaf tube’ by joining together the ends of a leaf, where they live and feed, moving to new leaves as necessary. Small skipper caterpillars usually pupate by June, with adult butterflies emerging in July, to spend their remaining days in tall grassland until the summer’s end in September.

## Genome sequence report

**Figure 1.**
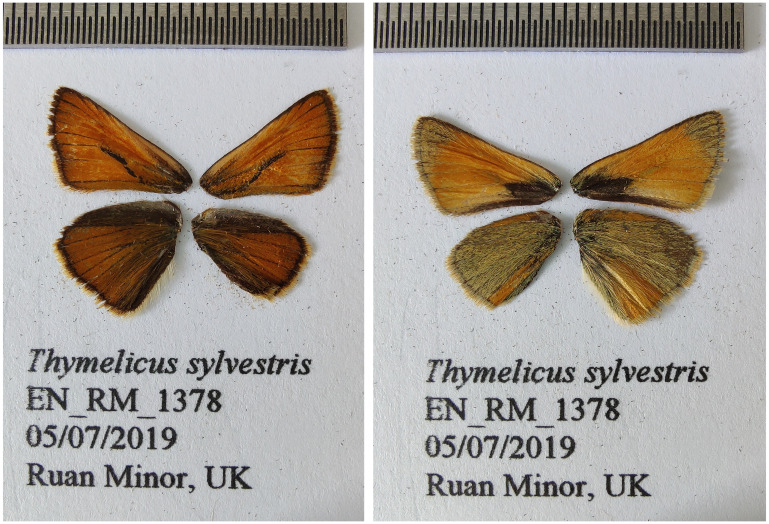
Fore and hind wings of the *Thymelicus sylvestris* specimen from which the genome was sequenced. Dorsal (left) and ventral (right) surface view of wings from specimen EN_RM_1378 (ilThySylv1) from Ruan Minor, Cornwall, UK, used to generate Pacific Biosciences and 10X genomics data.

The genome was sequenced from a single male *T. sylvestris* collected from Ruan Minor, Cornwall, UK (latitude 49.9942295, longitude -5.1974720) ( Figure 1). A total of 40-fold coverage in Pacific Biosciences single-molecule long reads and 63-fold coverage in 10X Genomics read clouds were generated. Primary assembly contigs were scaffolded with chromosome conformation Hi-C data. Manual assembly curation corrected 9 missing/misjoins and removed 3 haplotypic duplications, reducing the assembly size by 0.06% and scaffold number by 20.00%.

**Figure 2.**
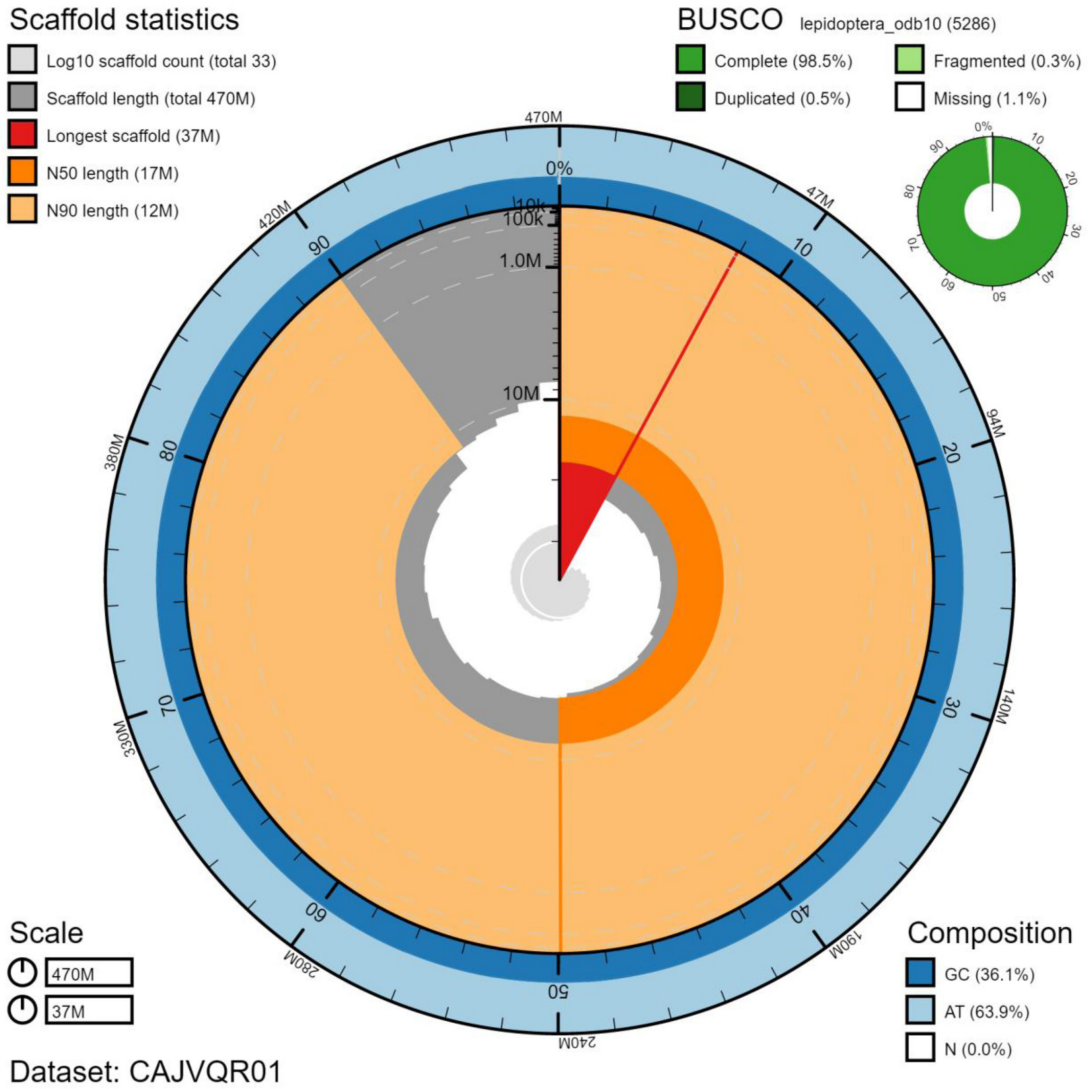
Genome assembly of *Thymelicus sylvestris*, ilThySylv1.1: metrics. BlobToolKit Snailplot shows N50 metrics and BUSCO gene completeness. The main plot is divided into 1,000 size-ordered bins around the circumference with each bin representing 0.1% of the 470,727,450 bp assembly. The distribution of chromosome lengths is shown in dark grey with the plot radius scaled to the longest chromosome present in the assembly (37,236,842 bp, shown in red). Orange and pale-orange arcs show the N50 and N90 chromosome lengths (17,253,319 and 11,732,099 bp), respectively. The pale grey spiral shows the cumulative chromosome count on a log scale with white scale lines showing successive orders of magnitude. The blue and pale-blue area around the outside of the plot shows the distribution of GC, AT and N percentages in the same bins as the inner plot. A summary of complete, fragmented, duplicated and missing BUSCO genes in the lepidoptera_odb10 set is shown in the top right. An interactive version of this figure is available at https://blobtoolkit.genomehubs.org/view/ilThySylv1.1/dataset/CAJVQR01/snail.

The final assembly has a total length of 471 Mb in 32 sequence scaffolds with a scaffold N50 of 17 Mb ( Table 1). Of the assembly sequence, 99.97% was assigned to 28 chromosomal-level scaffolds, representing 27 autosomes (numbered by sequence length), and the Z sex chromosome ( Figure 2– Figure 5; Table 2). The assembly has a BUSCO ( Simão *et al.*, 2015) v5.1.2 completeness of 98.5% (single 98.1%, duplicated 0.5%) using the lepidoptera_odb10 reference set. While not fully phased, the assembly deposited is of one haplotype. Contigs corresponding to the second haplotype have also been deposited.

**Table 1.** Genome data for *Thymelicus sylvestris*, ilThySylv1.1.

*Project accession data*	
Assembly identifier	ilThySylv1.1
Species	*Thymelicus sylvestris*
Specimen	ilThySylv1/EN_RM_1378 (genome assembly); ilThySylv2 (RNA-Seq); ilThySylv3 (Hi-C)
NCBI taxonomy ID	NCBI:txid272628
BioProject	PRJEB41953
BioSample ID	SAMEA7523279
Isolate information	Male, whole organisms
*Raw data accessions*	
PacificBiosciences SEQUEL II	ERR6608659
10X Genomics Illumina	ERR6363310-ERR6363313
Hi-C Illumina	ERR6363315
Illumina PolyA RNA-Seq	ERR6363314
*Genome assembly*	
Assembly accession	GCA_911387775.1
*Accession of alternate haplotype*	GCA_911387695.1
Span (Mb)	471
Number of contigs	46
Contig N50 length (Mb)	16.6
Number of scaffolds	32
Scaffold N50 length (Mb)	17.3
Longest scaffold (Mb)	21.0
BUSCO * genome score	C:98.5%[S:98.1%,D:0.5%],F:0.3%,M:1.1%,n:5286

*BUSCO scores based on the lepidoptera_odb10 BUSCO set using v5.1.2. C= complete [S= single copy, D=duplicated], F=fragmented, M=missing, n=number of orthologues in comparison. A full set of BUSCO scores is available at https://blobtoolkit.genomehubs.org/view/ilThySylv1.1/dataset/CAJVQR01/busco.

**Figure 3.**
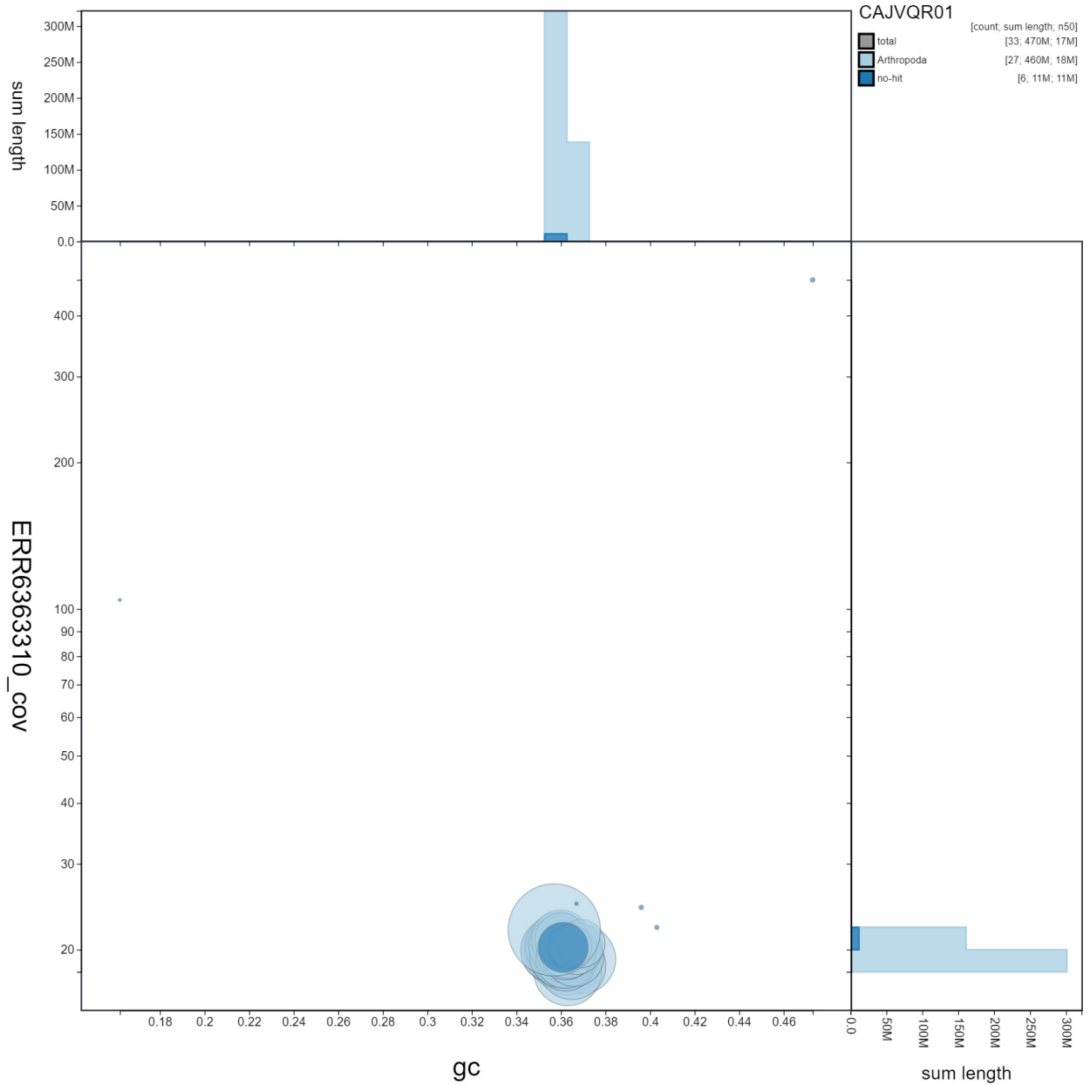
Genome assembly of *Thymelicus sylvestris*, ilThySylv1.1: GC coverage. BlobToolKit GC-coverage plot. Scaffolds are coloured by phylum. Circles are sized in proportion to scaffold length. Histograms show the distribution of scaffold length sum along each axis. An interactive version of this figure is available at https://blobtoolkit.genomehubs.org/view/ilThySylv1.1/dataset/CAJVQR01/blob.

**Figure 4.**
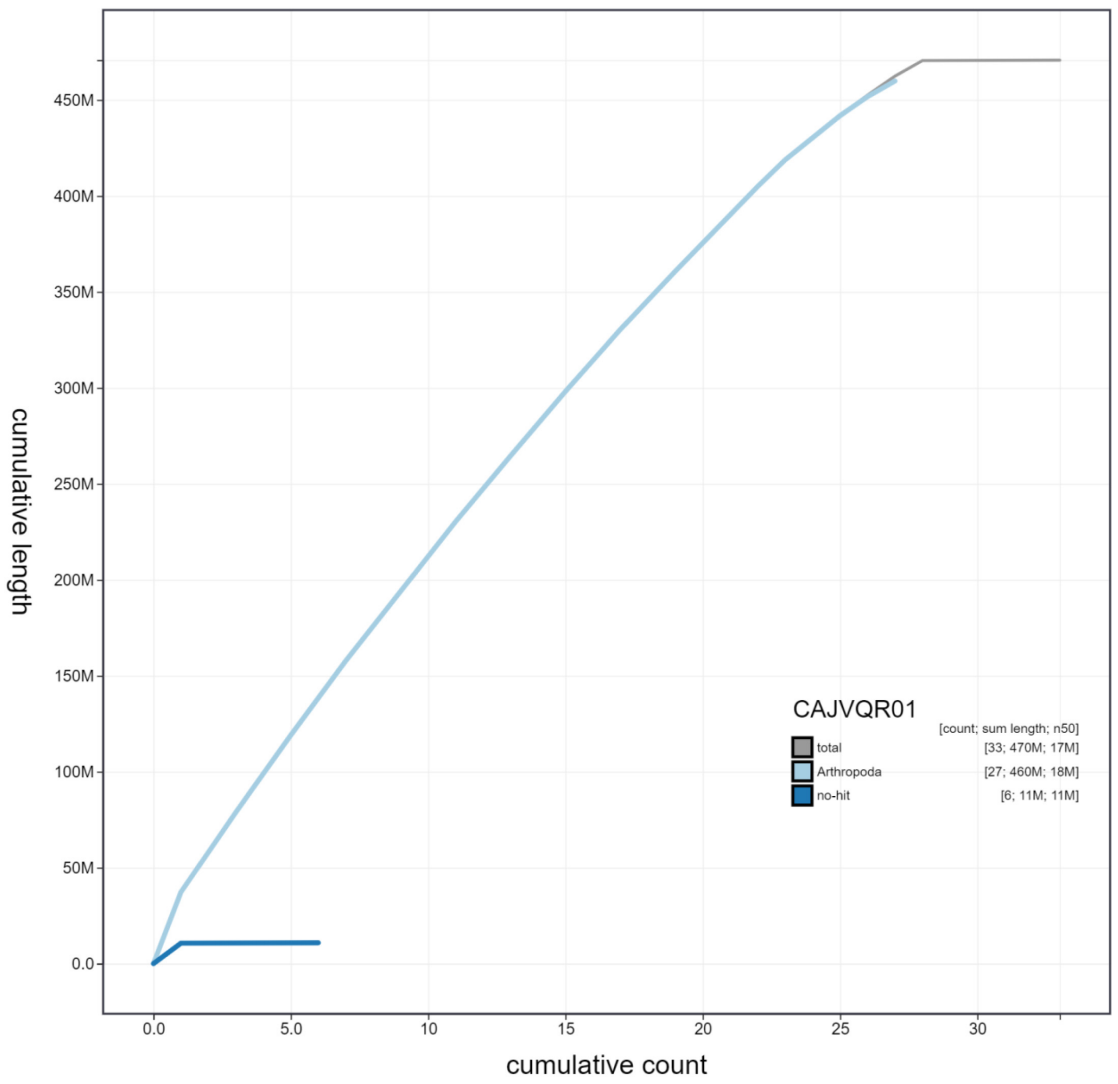
Genome assembly of *Thymelicus sylvestris*, ilThySylv1.1: cumulative sequence. BlobToolKit cumulative sequence plot. The grey line shows cumulative length for all scaffolds. Coloured lines show cumulative lengths of scaffolds assigned to each phylum using the buscogenes taxrule. An interactive version of this figure is available at https://blobtoolkit.genomehubs.org/view/ilThySylv1.1/dataset/CAJVQR01/cumulative.

**Figure 5.**
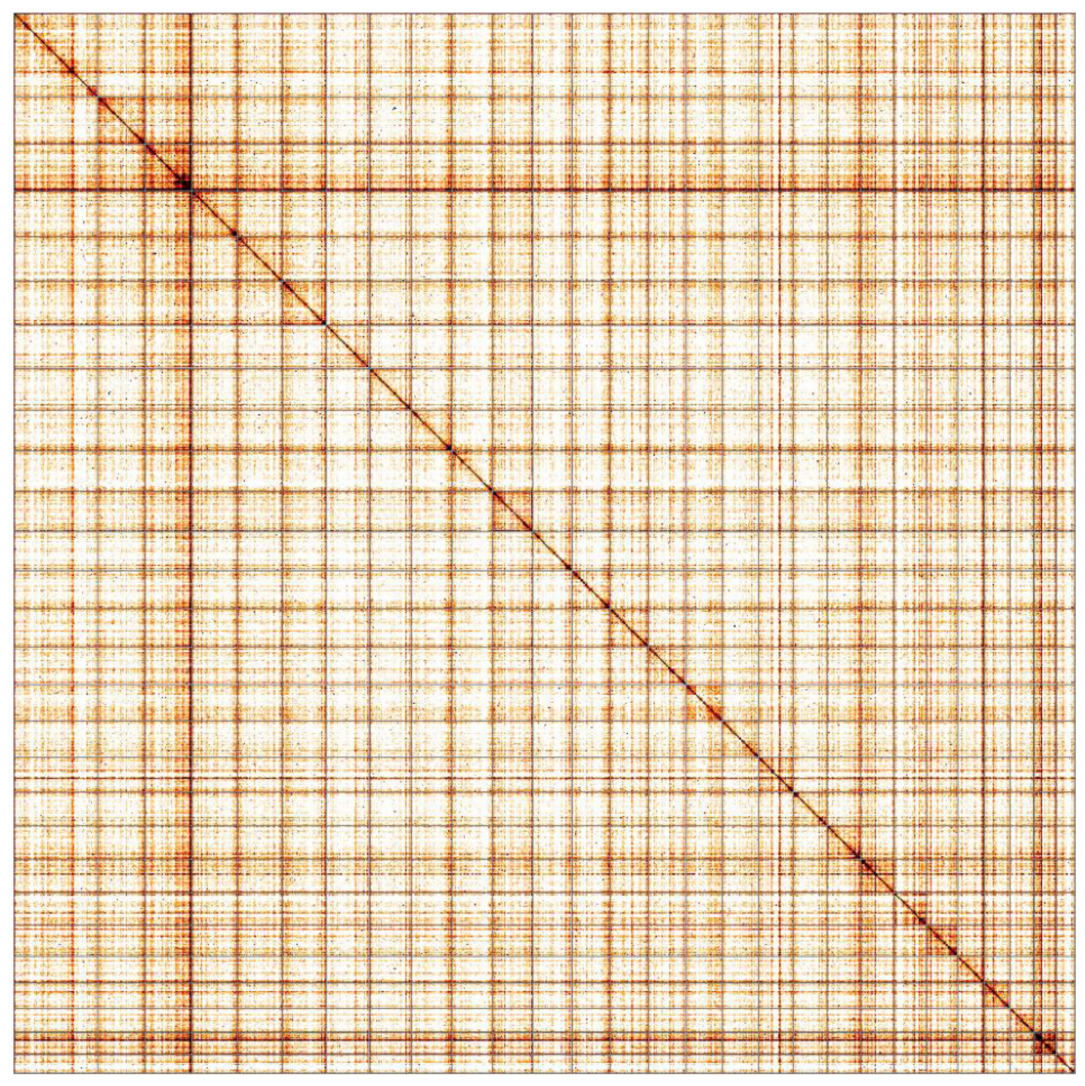
Genome assembly of *Thymelicus sylvestris*, ilThySylv1.1: Hi-C contact map. Hi-C contact map of the ilThySylv1.1 assembly, visualised in HiGlass. Chromosomes are arranged in size order from left to right and top to bottom.

**Table 2.** Chromosomal pseudomolecules in the genome assembly of *Thymelicus sylvestris*, ilThySylv1.1.

INSDC accession	Chromosome	Size (Mb)	GC%
OU426886.1	1	20.99	36.0
OU426887.1	2	20.74	36.9
OU426888.1	3	20.31	36.3
OU426889.1	4	19.76	36.3
OU426890.1	5	19.60	36.5
OU426891.1	6	19.09	35.7
OU426892.1	7	18.50	35.9
OU426893.1	8	18.16	35.9
OU426894.1	9	17.98	35.8
OU426895.1	10	17.90	36.0
OU426896.1	11	17.25	35.6
OU426897.1	12	17.04	35.8
OU426898.1	13	16.99	36.2
OU426899.1	14	16.61	36.0
OU426900.1	15	16.11	36.2
OU426901.1	16	16.10	35.9
OU426902.1	17	15.35	35.9
OU426903.1	18	15.05	36.0
OU426904.1	19	14.87	36.2
OU426905.1	20	14.87	36.6
OU426906.1	21	14.38	36.2
OU426907.1	22	13.89	36.3
OU426908.1	23	11.73	36.0
OU426909.1	24	11.38	36.7
OU426910.1	25	10.65	36.1
OU426911.1	26	9.84	36.9
OU426912.1	27	8.15	36.4
OU426885.1	Z	37.24	35.7
OU426913.1	MT	0.02	16.7
-	Unplaced	0.18	41.7

## Methods

### Specimen acquisition and nucleic acid extraction

Three male *T. sylvestris* (ilThySylv1, ilThySylv2 and ilThySylv3) specimens were collected from Ruan Minor, Cornwall, UK (latitude 49.9942295, longitude -5.1974720) using a net by Alex Hayward in May 2019. The samples were identified by the same individual and snap-frozen on dry ice.

DNA was extracted from the whole organism of ilThySylv1 (specimen ID: EN_RM_1378) at the Wellcome Sanger Institute (WSI) Scientific Operations core from the whole organism using the Qiagen MagAttract HMW DNA kit, according to the manufacturer’s instructions. RNA from whole organism tissue of ilThySylv2 was extracted in the Tree of Life Laboratory at the WSI using TRIzol, according to the manufacturer’s instructions. RNA was then eluted in 50 μl RNAse-free water and its concentration assessed using a Nanodrop spectrophotometer and Qubit Fluorometer using the Qubit RNA Broad-Range (BR) Assay kit. Analysis of the integrity of the RNA was done using the Agilent RNA 6000 Pico Kit and Eukaryotic Total RNA assay.

### Sequencing

Pacific Biosciences HiFi circular consensus and 10X Genomics Chromium read cloud sequencing libraries were constructed according to the manufacturers’ instructions. Poly(A) RNA-Seq libraries were constructed using the NEB Ultra II RNA Library Prep kit. Sequencing was performed by the Scientific Operations core at the Wellcome Sanger Institute on Pacific Biosciences SEQUEL II (HiFi), Illumina HiSeq X (10X) and Illumina HiSeq 4000 (RNA-Seq) instruments. Hi-C data were generated from head tissue of ilThySylv3 in the Tree of Life Laboratory using the Arima Hi-C+ kit and sequenced on an Illumina NovaSeq 6000 instrument.

### Genome assembly

Assembly was carried out with Hifiasm ( Cheng *et al.*, 2021). Haplotypic duplication was identified and removed with purge_dups ( Guan *et al.*, 2020). One round of polishing was performed by aligning 10X Genomics read data to the assembly with longranger align, calling variants with freebayes ( Garrison & Marth, 2012). The assembly was then scaffolded with Hi-C data ( Rao *et al.*, 2014) using SALSA2 ( Ghurye *et al.*, 2019). The assembly was checked for contamination and corrected using the gEVAL system ( Chow *et al.*, 2016) as described previously ( Howe *et al.*, 2021). Manual curation was performed using gEVAL, HiGlass ( Kerpedjiev *et al.*, 2018) and Pretext. The mitochondrial genome was assembled using MitoHiFi ( Uliano-Silva *et al.*, 2021). The genome was analysed and BUSCO scores generated within the BlobToolKit environment ( Challis *et al.*, 2020). Table 3 contains a list of all software tool versions used, where appropriate.

**Table 3.** Software tools used.

Software tool	Version	Source
Hifiasm	0.15	Cheng *et al.*, 2021
purge_dups	1.2.3	Guan *et al.*, 2020
SALSA2	2.2	Ghurye *et al.*, 2019
longranger align	2.2.2	https://support.10xgenomics.com/genome-exome/software/pipelines/latest/advanced/other-pipelines
freebayes	1.3.1-17-gaa2ace8	Garrison & Marth, 2012
MitoHiFi	2.0	Uliano-Silva *et al.*, 2021
gEVAL	N/A	Chow *et al.*, 2016
HiGlass	1.11.6	Kerpedjiev *et al.*, 2018
PretextView	0.2.x	https://github.com/wtsi-hpag/PretextView
BlobToolKit	2.6.4	Challis *et al.*, 2020

## Data availability

European Nucleotide Archive: Thymelicus sylvestris (small skipper). Accession number PRJEB45673; https://identifiers.org/ena.embl/PRJEB45673.

The genome sequence is released openly for reuse. The *T. sylvestris* genome sequencing initiative is part of the Darwin Tree of Life (DToL) project. All raw sequence data and the assembly have been deposited in INSDC databases. The genome will be annotated using the RNA-Seq data and presented through the Ensembl pipeline at the European Bioinformatics Institute. Raw data and assembly accession identifiers are reported in Table 1.

## Author information

Members of the Darwin Tree of Life Barcoding collective are listed here: https://doi.org/10.5281/zenodo.5744972.

Members of the Wellcome Sanger Institute Tree of Life programme are listed here: https://doi.org/10.5281/zenodo.5744840.

Members of Wellcome Sanger Institute Scientific Operations: DNA Pipelines collective are listed here: https://doi.org/10.5281/zenodo.5746904.

Members of the Tree of Life Core Informatics collective are listed here: https://doi.org/10.5281/zenodo.5743293.

Members of the Darwin Tree of Life Consortium are listed here: https://doi.org/10.5281/zenodo.5638618.
