# Supplementary material for: The genome sequence of the Small Skipper, Thymelicus sylvestris (Poda, 1761)
Source: Wellcome Open Res. 2025 Sep 26;7:35. Originally published 2022 Feb 1. [Version 2] doi: 10.12688/wellcomeopenres.17577.2 (PMC12595296; doi:10.12688/wellcomeopenres.17577.2)
Supplement: Peer review history

# Reviewer report: Reviewer response for version 2

**DOI:** 10.21956/wellcomeopenres.27568.r134447

**Published:** 2026-01-06

**Authors:** Xueyan Li

**Affiliations:** 1 Kunminng Institute of Zoology, Chinese Academy of Sciences, Kunming, China

This paper “The genome sequence of the small skipper, *Thymelicus sylvestris *(Poda, 1761)” have now been improved well. I think it is ok now.

Are sufficient details of methods and materials provided to allow replication by others?

Partly

Is the rationale for creating the dataset(s) clearly described?

Yes

Are the datasets clearly presented in a useable and accessible format?

Yes

Are the protocols appropriate and is the work technically sound?

Yes

Reviewer Expertise:

Insect genome and evolution

I confirm that I have read this submission and believe that I have an appropriate level of expertise to confirm that it is of an acceptable scientific standard.

# Reviewer report: Reviewer response for version 2

**DOI:** 10.21956/wellcomeopenres.27568.r138439

**Published:** 2025-11-27

**Authors:** Vlad Dinca

**Affiliations:** 1 "Grigore Antipa" National Museum of Natural History, Bucharest, Romania

In this data note, the authors have successfully generated a chromosome-level genome assembly of a male *Thymelicus sylvestris *(Lepidoptera: Hesperiidae). The mitochondrial genome has been assembled as well.

 Although I am not an expert in several of the methodological approaches used, as far as I can tell, the manuscript is technically sound and uses methodologies and pipelines that are fairly well-established through use by the Wellcome Sanger Institute.

 The genome appears to be of high quality and scaffolded into 28 chromosomal pseudomolecules (including the Z sex chromosome).

 A few additional comments are included below.

 I would say it is absent from most of Scandinavia, as it is basically present in Denmark.

*Holcus lanatus* does indeed seem to be one of the preferred host plants, but several others have been reported across Europe. Thus, it may be good to list a few other species on which larvae have been reported (see for example Clarke 2024 -

https://doi.org/10.1002/ece3.10834).

 The species can be on wing earlier, at least during May as well. Please update this. The specimen analysed in this note is later mentioned to have been collected in May 2019.

 Please also update the mention that the adults emerge in July. Across Europe, depending on location, the flight time can start even at the end of April and will usually end in August. The “core” of the flight time is usually between May-July.

 It may also be useful adding 1-2 sentences about what is currently known about the genetic structure of this species across Europe. See for example Hinojosa et al. (2019 -

https://doi.org/10.1111/mec.15153).

Are sufficient details of methods and materials provided to allow replication by others?

Yes

Is the rationale for creating the dataset(s) clearly described?

Yes

Are the datasets clearly presented in a useable and accessible format?

Yes

Are the protocols appropriate and is the work technically sound?

Yes

Reviewer Expertise:

Lepidoptera taxonomy, phylogeography, conservation.

I confirm that I have read this submission and believe that I have an appropriate level of expertise to confirm that it is of an acceptable scientific standard.

# Reviewer report: Reviewer response for version 2

**DOI:** 10.21956/wellcomeopenres.27568.r136729

**Published:** 2025-11-08

**Authors:** Xiaoling Fan

**Affiliations:** 1 Department of Entomology, College of Plant Protection, South China Agricultural University, Guangzhou, China

This manuscript presents a chromosomal-level genome assembly for the Small Skipper, *Thymelicus sylvestris*, achieved through the integration of PacBio HiFi, 10X, RNA-seq, and Hi-C data from fresh specimens. The assembly's exceptional continuity (scaffold N50 of 17.25 Mb) and completeness (98.5% BUSCO score) establish it as a foundational and highly reliable resource for future research in skipper butterfly genomics.

Are sufficient details of methods and materials provided to allow replication by others?

Yes

Is the rationale for creating the dataset(s) clearly described?

Yes

Are the datasets clearly presented in a useable and accessible format?

Yes

Are the protocols appropriate and is the work technically sound?

Yes

Reviewer Expertise:

systematics

I confirm that I have read this submission and believe that I have an appropriate level of expertise to confirm that it is of an acceptable scientific standard.

# Reviewer report: Reviewer response for version 1

**DOI:** 10.21956/wellcomeopenres.19436.r50767

**Published:** 2022-06-09

**Authors:** Xueyan Li

**Affiliations:** 1 Kunminng Institute of Zoology, Chinese Academy of Sciences, Kunming, China

This paper “The genome sequence of the small skipper, *Thymelicus sylvestris *(Poda, 1761)“ assembled the chromosomal-level genome of *Thymelicus sylvestris*. Due to the HiFi, 10X Genomics, Hi-C Illumina and RNA-seq, the assembly quality is relatively high. The high-quality reference genomes of more butterfly species will be helpful to investigate the evolution of butterfly diversity.

 In general, this paper is well written with a concise description. Nevertheless, the authors didn’t provide genome annotation (repeat and genes), which, if unavailable, will limit the use of the data, especially for non-bioinformatic specialists. So, please provide genome annotation information.

 Several revisions are needed for the data.

- First, how many chromosomes for this species, please describe it and cite the related literature.
- Second, how the authors identified the Z sex chromosome?

Minor revisions

- Background: “The skippers are named for their characteristic quick, darting flight.“ to “The skippers are named for their characteristically quick, darting flight.”.

Are sufficient details of methods and materials provided to allow replication by others?

Partly

Is the rationale for creating the dataset(s) clearly described?

Yes

Are the datasets clearly presented in a useable and accessible format?

Yes

Are the protocols appropriate and is the work technically sound?

Yes

Reviewer Expertise:

Insect genome and evolution

I confirm that I have read this submission and believe that I have an appropriate level of expertise to confirm that it is of an acceptable scientific standard, however I have significant reservations, as outlined above.

# Response

**Published:** 2025-09-23

**Authors:** Tree of Life Team Sanger

**Affiliations:** Wellcome Trust Sanger Institute, UK

Thank you for reviewing this data note. We have made changes to the article in Version 2 and our responses to your comments are given below.

- “Nevertheless, the authors didn’t provide genome annotation (repeat and genes), which, if unavailable, will limit the use of the data, especially for non-bioinformatic specialists. So, please provide genome annotation information.”

**Response:** Genome annotation is not part of our assembly pipeline but is done externally by Ensembl at the European Bioinformatics Institute. We have added information and a link to the Ensembl page for the genome annotation, which has been supplied since version 1 of the data note was published.

- First, how many chromosomes for this species, please describe it and cite the related literature.

**Response: **Thank you for this suggestion. We have added information to the background section about the related literature. Previously cytology done in 1960 found a chromosome count of *n* = 27. Although this was the expected chromosome count for our assembly, we found an additional chromosome, and the data did not support further joins being made.

- Second, how the authors identified the Z sex chromosome?

**Response: **The Z chromosome is identified based on synteny to related species and the presence of conserved BUSCO genes.

# Reviewer report: Reviewer response for version 1

**DOI:** 10.21956/wellcomeopenres.19436.r48488

**Published:** 2022-02-21

**Authors:** Francois Olivier Hebert

**Affiliations:** 1 Cannabis Innovation and Research Center, Université de Moncton, Moncton, BC, Canada; 2 CR-CHUM, Université de Montréal, Montréal, Canada

**General comments:**

 This short and well written manuscript presents the genome sequence of a male Thymelicus sylvestris (small skipper) sample in the context of the Darwin Tree of Life project. The genome assembly in this work was generated with a combination of PacBio HiFi reads and additional 10X sequencing from a single male individual, and RNAseq and HiC sequencing for genome improvement. They report a final genome sequence of 471 Mb, which is 98.5% complete according to its BUSCO score. Although I am not an expert per say in entomology or specifically in skipper biology, I found this piece of work interesting, written in a straightforward manner and in a simple form. This work is totally worth publishing because it provides a very high-quality reference genome sequence for a species that was still lacking from public databases. It allows the research community to expand its general knowledge on butterfly genomics and it also provides additional data that can be used in the future to perform comparative analyses and better understand insect genome evolution on a broader sense. The authors did a good job at generating a complete and highly contiguous/complete genome sequence.

 Although this manuscript is very clear and the assembly of high quality (props to the team for the cleaness of this assembly), I would have liked to learn a little bit more about the reason(s) behind this genome sequence, i.e., the “what we know”, “what we don’t know”, and what it is that this genome sequence potentially brings to the table to better understand what we don’t know about this species or its related taxa. Just to put the work into context. Maybe on the form of just a sentence on what the ultimate goal of the Darwin Tree of Life project is? And maybe a little bit more details in the methods in terms of command lines and scripts used to run the programs (which would augment the repeatability of this nice work). But apart from that, great work! Down there are some comments that could increase the quality of the paper if addressed by the authors:

**Specific comments:**

- Suggestion: just a tiny minor detail, but maybe specify in the abstract that it’s 27 chromosomes + Z chromosome, for a total of 28 chromosomes? It is well mentioned in the main text, but it could be phrased differently in the abstract so that it is does not create any ambiguity when just reading the abstract by itself.

- Impressive quality in terms of BUSCO score: little duplicated BUSCO genes and 98% single is very good, especially in animal species with (typically) large quantities of repeated elements.

- On that note, is there a report somewhere of the percentage of repeats in this assembly? Or maybe this information is not relevant in the context of the DToL project, but I would have liked to have a sense of how repeated that genome is. I presume that there is no gene annotation report so far? If yes, this would be valuable to provide details on the numbers of genes, gene densities on the chromosomes, and other relevant functional annotation metrics.

- Written in the “general comments” section, but again here: in the introduction, I think it would be interesting for the readership to learn a little bit more about the reasons behind this work. Why produce a genome sequence of this species in particular? How is it part of a larger initiative with a particular goal, for instance here, sequencing numerous species with a super large comparative genomics perspective in mind (i.e., understanding the evolutionary genomics / mapping the genome of all animals living in a common geographic location)? In other words, a brief rationale justifying the dataset.

- In the section Genome sequence report, first paragraph, it says that the genome was sequenced from a single individual male, but later in the Methods section, the authors report the capture of three samples. In Table 1, it looks like these three samples were used to generate different libraries of different sequencing technologies, all involved in the genome assembly method. I was thinking that the authors could specify this detail in the first paragraph of the “Genome sequence report” section when they mention the different technologies used in their study, i.e., which technology was used to sequence which sample (and maybe the reason why three samples?)? Perhaps I’m missing something here in terms of assembly strategy, but in the end, if 3 different samples were used, the final genome assembly is not entirely from a single individual… or is it? Why not use these 3 technologies on the same sample? I know that extracting enough DNA material in a single individual insect can sometimes be quite difficult (low yields), so maybe this is the reason why the authors chose to use three samples? Just a short clarification on that note would elevate the quality of the paper… to my humble opinion.

- I am by no means an expert in Lepidoptera, although I’ve assembled a lepidopteran genome in the past, I am wondering, out of curiosity, how many chromosomes are expected in this species specifically? Is it 28 or 31? I know chromosome numbers are quite variable in Hesperidae, but isn’t it supposed to be 31 usually (or ancestrally?)? Maybe the authors could briefly comment on that topic in the manuscript, i.e., if this number of chromosomes is expected or not? If there are 98% complete single BUSCO group, we can presume that no chromosomes are missing though, but maybe there are 28 chromosomes assembled here (out of 32 scaffolds?), but there should be 30 or 31 and the rest is in the “unanchored” contigs and remaining scaffolds? Could the authors, please, clarify this?

- Data accessibility: I respect so much the fact that everything from this manuscript seems to be transparent and open source. Maybe I missed something in the data provided with this work, but is there a mention somewhere of the scripts or command lines used by the authors to produce this assembly? Or maybe all the programs were used with default values (which is sometimes recommended anyways when reading these tools’ manual)? The protocols are appropriate, and the work is technically sound, but it lacks a little bit of method details to be repeatable.

Are sufficient details of methods and materials provided to allow replication by others?

Partly

Is the rationale for creating the dataset(s) clearly described?

Partly

Are the datasets clearly presented in a useable and accessible format?

Yes

Are the protocols appropriate and is the work technically sound?

Yes

Reviewer Expertise:

Functional genomics, genome assembly, RNAseq, physiology

I confirm that I have read this submission and believe that I have an appropriate level of expertise to confirm that it is of an acceptable scientific standard.

# Response

**Published:** 2025-09-23

**Authors:** Tree of Life Team Sanger

**Affiliations:** Wellcome Trust Sanger Institute, UK

Thank you for reviewing this data note. We have made the following changes in Version 2, which we hope will provide more clarity.

- We have clarified the chromosome count in the abstract as you recommend. The chromosome map in Figure 3 is now labelled, which we hope will improve the data note.
- As the genome has now been annotated, we have provided a summary of the annotated and a link to the information on Ensembl.
- We have added a table showing the specimen and sequencing information. Three different individuals were used for PacBio HiFi, Illumina Hi-C and RNA-seq. The genome is that of only one individual, the one used for PacBio sequencing.
- We have added a paragraph on the expected chromosome count for the family of skippers, and we compare the chromosome number recovered here to the expected count. There are no additional chromosomes in the unassembled scaffolds, as the sequence is almost completely assembled.
- The main rationale for sequencing this species is that it is on the Darwin Tree of Life list, and we have
- We have added a link to the software repository for Tree of Life assemblies in the Data availability section.

The Tree of Life team
